# Human Recombinant FSH and Its Biosimilars: Clinical Efficacy, Safety, and Cost-Effectiveness in Controlled Ovarian Stimulation for In Vitro Fertilization

**DOI:** 10.3390/ph13070136

**Published:** 2020-06-27

**Authors:** Loredana Bergandi, Stefano Canosa, Andrea Roberto Carosso, Carlotta Paschero, Gianluca Gennarelli, Francesca Silvagno, Chiara Benedetto, Alberto Revelli

**Affiliations:** 1 Department of Oncology, University of Torino, Via Santena 5 bis, 10126 Torino, Italy; francesca.silvagno@unito.it; 2 Gynecology and Obstetrics 1, Physiopathology of Reproduction and IVF Unit, Department of Surgical Sciences, S. Anna Hospital, University of Torino, Via Ventimiglia 3, 10126 Torino, Italy; s.canosa88@gmail.com (S.C.); andrea88.carosso@gmail.com (A.R.C.); carlotta.paschero@libero.it (C.P.); gianluca.gennarelli@unito.it (G.G.); chiara.benedetto@unito.it (C.B.); alberto.revelli@unito.it (A.R.)

**Keywords:** recombinant human follicle-stimulating hormone (r-hFSH), r-hFSH biosimilars, controlled ovarian stimulation, in vitro fertilization

## Abstract

Exogenous human follicle-stimulating hormone (hFSH), either derived from extraction and purification from the urine or obtained by recombinant technology in the form of follitropin *α*, *β* and *δ* (rFSH), has been used for decades in the treatment of infertility. The main applications of FSH treatment in the woman have been, and still are, ovulation induction in oligo-anovulatory subjects, and stimulation of the development of a cohort of follicles in patients undergoing controlled ovarian stimulation (COS) for in vitro fertilization (IVF). In the last years, two biosimilars of follitropin alfa, rFSH compounds structurally and functionally similar to the originator, have been approved and marketed for clinical use in Europe. Moreover, some other rFSH biosimilars are currently under investigation. The objective of this article is to review the available evidences comparing the efficacy, safety, and cost-effectiveness of rFSH follitropin alpha originator with its biosimilars, discussing the clinical trials that allowed biosimilars to get registration and marketing authorization.

## 1. Introduction

Human follicle-stimulating hormone (hFSH) is produced by the anterior pituitary gland and plays a key role in the regulation of fertility in both men and women. Medications containing hFSH have been used for decades to treat infertile women with anovulatory cycles or to accomplish controlled ovarian stimulation (COS) in patients undergoing in vitro fertilization (IVF) [1]. Exogenous hFSH has also been used for the treatment of male hypogonadotrophic hypogonadism [2].

Since the early years of assisted reproductive technology (ART), exogenous hFSH has been administered to stimulate follicular growth both when mono-follicular development was desired (coupled to Intrauterine Insemination or timed intercourse) or when multi-follicular growth was required to obtain multiple oocytes, as in IVF [3]. Actually the number of retrieved eggs is a major variable affecting IVF success rate [4], and therefore COS by FSH medications is considered to play a pivotal role in determining the effectiveness of IVF treatment [5]. 

This review focuses on the different forms of recombinant FSH (rFSH) and on their biosimilar commercially available preparations, comparing their clinical efficacy, safety, and cost-effectiveness when used to treat women undergoing COS for IVF. Differently from other reviews on the topic, it provides not only a thorough update of the most recent studies published in peer-reviewed journals and of completed/ongoing clinical trials with FSH biosimilars in infertile women undergoing IVF, but also a detailed discussion of the design, results and power calculation of registration studies. This point is of prominent value to properly understand the difference with biosimilarity and bioequivalence when the most important IVF outcomes are concerned.

**Figure 1:**
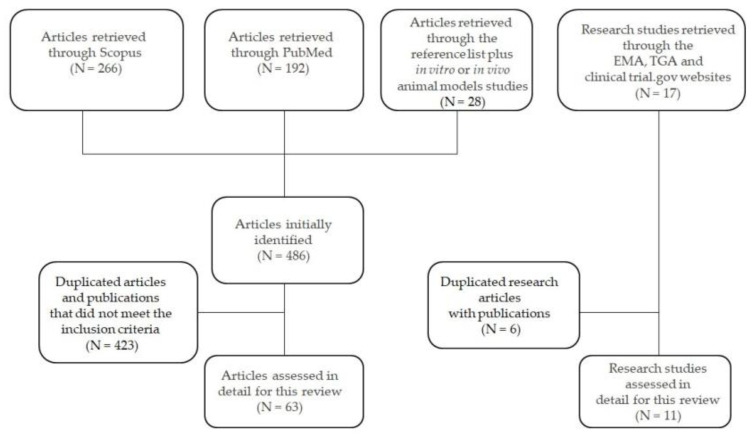
Search strategy for identifying scientific publications and clinical studies for this comprehensive review paper. Abbreviations: EMA, European Medicines Agency, and TGA, Therapeutic Good Administration.

Electronic searches were performed using Scopus and PubMed from January 2006 to May 2020 selecting humans as species, classical article, review and medicine, biochemistry, genetics and molecular biology as subject area in Scopus, and humans as species, classical article, review, clinical trial—Phase I, II, III, IV—in PubMed. Abstracts, case reports, conference presentations were excluded. To identify all relevant published studies, we combined the following medical subject headings (MeSH) terms or keywords: “controlled ovarian stimulation” OR “in vitro fertilization” OR “anovulatory cycles” AND “human recombinant FSH” OR “follitropin-alpha“ OR “human FSH recombinant biosimilar”. All publications were in English and limited to human subjects, with the only exception of 18 additional papers concerning in vitro and in vivo animal model studies retrieved from the reference lists of some selected articles. Indeed the reference list of all retrieved articles was also reviewed and additional 10 articles were considered even if they were published before 2006. At least 50% of the references had to be articles published within the last 5 years, as requested by the editorial rules of *Pharmaceuticals*; for this reason, most studies comparing follitropin *α* vs. *β* and studies about corifollitropin *α* were excluded as they were published earlier. A total of 486 publications were retrieved through the research databases. After excluding duplicated articles and publications that did not meet inclusion criteria, 63 articles remained, and were reviewed together with 11 research studies retrieved through the European Medicines Agency (EMA), the Therapeutic Good Administration (TGA) and the clinicaltrials.gov websites using the following keywords: FSH biosimilar, human and female infertility (Figure 1). 

## 2. Structure of hFSH 

The human gonadotropins follicle-stimulating hormone (hFSH), luteinizing hormone (hLH) and chorionic gonadotropin (hCG) are complex heterodimeric glycoprotein hormones composed of two non-covalently linked protein subunits, the *α*- and *β*-chains. 

The *α*-subunit is identical in hFSH, hLH, and hCG, contains 92 amino acids and carries N-linked oligosaccharides added to the asparagine (Asn) residues 52 and 78. The *β* subunit is slightly different in the three gonadotropins, and confers the specificity of action. In hFSH, the *β* subunit is composed of 111 amino acids and it is responsible for proper folding, assembly, receptor binding specificity and biological properties. Notably, the hFSH-*β* subunit contains two glycosylation sites: Asn^7^ and Asn^24^. This entails four sites of glycosylation that confer different molecular weight to the hFSH: a fully glycosylated form of FSH, indicated as hFSH^24^ (M_r_ 24,000), the two di-glycosylated forms hFSH^21^ (M_r_ 21,000) and hFSH^18^ (M_r_ 18,000), carrying glycosylations at the Asn^7^ and Asn^24^, respectively, and the hFSH^15^ (M_r_ 15,000), lacking glycosylation on the *β*-subunit [6]. Moreover, as each branch of the oligosaccharides may or may not terminate in a negatively charged sialic acid residue, different isoforms with different isoelectric points, plasma half-life (ranging from 3 to 4 h) and bioactivity may be generated [7]. Increased sialylation enhances hFSH metabolic stability, and thus lengthens its half-life by decreasing both glomerular filtration and clearance by sialoglycoprotein receptors in the liver, which is the major site for gonadotropin clearance [3,8,9]. 

The profile of hFSH isoform distribution is significantly dependent on its source, on the gender, on age and, in women, on the phase of the ovarian cycle [10]. The ratio of hFSH^21^/hFSH^24^ in the pituitary changes from hFSH^21^-dominance in women aged 21–24 to a roughly balanced proportion between 39 and 41 years, to hFSH^24^-dominance in women aged 55–81 [11]. Moreover, the composition of serum hFSH isoforms exhibits characteristic fluctuations during the menstrual cycle; in the early follicular phase acidic isoforms predominate, gradually shifting towards preferentially less acidic isoforms during the mid-follicular and late follicular phases, as ovulation approaches [9,10]. The less-acidic isoforms exhibit a shorter serum half-life as they have a faster clearance than the acidic isoforms. During the follicular recruitment, taking place in the early follicular phase, a longer half-life of hFSH ensures a higher efficacy in recruiting the pool of small antral follicles that will grow that month. Differently, during the more advanced phase of follicular development, when a dominant follicle has already been selected, isoforms with a shorter half-life are preferable to ensure the final maturation of granulosa cells and their optimal support to the cytoplasmic maturation of the oocyte [3]. 

The hypoglycosylated hFSH is much more biologically active than fully glycosylated hFSH [12], and highly acidic hFSH isoforms are produced in higher concentration after the menopause than during the reproductive age. This suggests that glycoform composition of circulating hormones is dynamic and has a physiological role [13], thus modulating the hormone-receptor structural interaction and the downstream signaling [14]. Indeed, it is reported that pituitary hFSH^21/18^ exhibits a 9- to 20-fold higher hFSH receptor-binding activity and occupies twice as many receptors than hFSH^24^ [11]. 

## 3. hFSH as a Medication: Historical Background 

In the history of human infertility therapy, the availability of drugs inducing ovulation in oligo-anovulatory patients or promoting the synchronous growth of a cohort of follicles in order to obtain several oocytes to be in vitro fertilized represented an enormous progress. The use of gonadotropins for the treatment of infertility began in the 1930s, following the discovery of FSH and LH. Early preparations were obtained from animal sources—e.g., the pregnant mare serum—or from extracts of the human pituitary gland taken post-mortem [15]. 

Subsequently, the human menopausal gonadotropin (hMG), extracted from the urine of women in menopausal age and containing a mixture of 1:1 FSH:LH activity together with miscellaneous urinary proteins, proved to be effective for ovarian stimulation and generally well tolerated [16]. The use of urinary gonadotropins was extended to women undergoing IVF treatment in the early 1980s, and the development of medications containing human gonadotropins continued, with the objective of improving their purity and providing a product with pure hFSH, free of hLH and other urine-derived contaminants. Indeed, different products of urinary derivation became available, such as urofollitropin (u-hFSH) in 1983, which had a FSH purity of approximately 5%, and highly purified urofollitropin (HP-u-hFSH) in 1993, with an FSH purity of approximately 95%. In more recent years, another urine-derived pharmaceutical gonadotropin, the highly purified HMG (HP-hMG), with a total gonadotropin content of approximately 70%, was developed [3]. In the early 1990s recombinant human FSH (r-hFSH) was obtained, and was widely recognized as a further progress in hFSH pharmacology, having higher purity, more intense specific activity, and superior efficacy in terms of pregnancy rate in in vitro fertilization cycles when compared with u-hFSH [17]. Urinary hFSH and hMG preparations, being extracted from post-menopausal urine, contain mostly hFSH^24^ [6]. Lombardi et al. showed a predominance of highly sialylated, highly branched glycans in u-hFSH as compared to r-hFSH, implying a weaker effect of u-hFSH on steroidogenesis in rodent cell lines; this observation suggests that differences in purity and molecular structure of medications are likely to be reflected in different biological properties that relevantly affect clinical results [10]. 

The composition of the recombinant hormone depends on manufacturing technology; briefly, the preparation of r-hFSH was obtained as follows: genes coding for *α*- and *β*-subunits of hFSH were transfected through plasmid expression into mammalian cells, the Chinese hamster ovary (CHO) cells [7], which are able to perform the post-translational step of hormonal production, protein folding and glycosylation [18]. Next, crude CHO cell culture supernatants were processed by using five chromatographic purification steps and ultrafiltration. The complete amino acid sequence of the *α*- and *β*-subunits of r-hFSH were determined by automated sequencing, while the structure of the carbohydrate chains was identified by glycan mapping. 

In the early 2000s the manufacturing process of r-hFSH was further improved in order to increase the specific activity and ensure a consistent isoform profile. The calibration of the final product was accomplished through a new method, the filled-by-mass process, that offers the opportunity to ensure improved batch-to-batch consistency, to guarantee that the r-hFSH dose is independent from any variation associated with the bioassay, and hence to standardize the ovarian stimulation and response [19]. Indeed, the consistency of the ovarian response to r-hFSH is a key issue; for clinicians performing IVF, the very low variability in the r-hFSH content between batches is a major advantage as it allows to finely tuning r-hFSH stimulation. The potential benefits of an optimal, individualized r-hFSH dose are the increased effectiveness and safety of COS, as well as the minimization of the risk of cycle cancellation for hypo- or of hyper-response [19].

## 4. Recombinant Human FSH (r-hFSH) 

Currently, there are different r-hFSH products on the market: (a) follitropin *α* (Gonal-F^®^, Merck KgaA, Darmstadt, Germany), available in Europe since 1995 and in USA from 2004 [20], (b) follitropin *β* (Puregon^®^, MSD, Darmstadt, Germany), marketed in Europe from 1996 [21] and in USA from 2004 (Follistim AQ, Merck KgaA, Darmstadt, Germany) [22], and (c) follitropin *δ* (Rekovelle^®^, also named FE 999049, Ferring Pharmaceuticals, St. Prex, Switzerland), recently developed and produced using human fetal retinal cells [23,24]. Follitropin *α*, *β* and *δ* have the same amino acid sequence; nevertheless, they differ in glycosylation, composition of sialic acid residues and isoelectric coefficients: follitropin *α* is more acidic than follitropin *β*, resulting in slightly different biological activity, half-life and metabolic clearance. Follitropin *α* and *β* have only *α*^2,3^-linked sialic acid, as 2,6-linked sialic acid is absent in CHO-derived r-hFSH; differently, follitropin *δ* includes tri- and tetra-sialylated glycans, has *α*^2,3^- and *α*^2,6^-linked sialic acid content, different sugars (such as *N*-acetylgalactosamine), and carries additional linkages among carbohydrates (such as bisecting *N*-acetylglucosamine and antennary fucose) [25,26]. 

Preclinical data from animal models showed that the currently available r-hFSH medications have no teratogenic, mutagenic or clastogenic effects, an evidence that was further supported by a series of trials and meta-analysis regarding the safety and effectiveness of r-hFSH for women undergoing IVF [16,27,28,29], including poor responder patients of advanced age and women with polycystic ovary syndrome (PCOS) [30]. Pharmacokinetic studies showed that r-hFSH is no more detectable in the woman’s blood by the time of embryo implantation, and no detrimental effects on the fetus after accidental exposure to r-hFSH in early pregnancy have been reported; moreover, r-hFSH is not believed to increase the risk of abortion or affect birthweight [31].

Follitropin *δ* received its marketing license in Europe in 2016 [32] and in Australia in 2017 [33]; nonetheless, it remains under additional clinical monitoring. In vitro, follitropin *δ* was observed to be equivalent to follitropin *α* when tested in cell-free FSH-receptor binding assays performed in stably transfected with the human FSH receptor human embryonic kidney cells and in human granulosa cells [26]. However, in rat models, follitropin *δ* was shown to have different pharmacokinetic and pharmacodynamic profiles, a quicker clearance, and a lower apparent potency. Consequently, follitropin *δ* cannot be dosed according to bioactivity or specific bioactivity in biological assays, as other follitropins are, and it is instead dosed by mass [26]. 

In order to overcome the relatively short biological half-life of r-hFSH, which requires daily administration, a long-acting form of r-hFSH, corifollitropin *α* (CF*α*, also named Elonva^®^; MSD, Readington, NJ, USA), was produced in transfected CHO cells [34]. This chimeric molecule includes the sequence encoding the C-terminal extension of hCG beta subunit (hCG-*β*), bearing four O-linked glycosylation sites and providing extended half-life of approximately 65 h [35], added to the FSH *β*-subunit. As a result of the longer serum half-life, a single injection of CF*α* can replace daily FSH injections for the first 7 days of COS, sustaining multiple follicular development as required for IVF. In primary cultures of human granulosa cells, CF*α* was demonstrated to maintain the specific actions of follitropin alpha, being even more potent in increasing aromatase gene expression inducing estrogen synthesis [36]. 

Another rFSH is under testing and is not yet marketed; it is called follitropin ε (FSH-GEX; Glycotope, Berlin, Germany) and is produced using a human blood cell line derived from myeloid leukemia cells [37]. Follitropin ε shows a high content of bisecting N-acetlyglucosamine, a high antennarity and a high degree of sialylation, in particular after enrichment of the acidic isoforms. Differently from follitropins *α* and *β*, follitropin ε is highly fucosylated and has a ratio of 2,3 to 2,6 sialylation of about 1:1, whereas follitropin *α* and *β* do not have any bisecting N-acetylgalactosamines or 2,6 sialylation [34]. In phase I studies, follitropin ε and follitropin *α* had similar pharmacokinetics, whereas pharmacodynamic activity (measured considering follicle growth and serum inhibin B secretion by granulosa cells) was higher with follitropin ε than with follitropin *α* [37,38].

From a clinical point of view, follitropin *α* and *β* were repeatedly shown to be equally effective for the use in COS for IVF. Actually, for about 25 years, they have been the only r-hFSH preparations available for clinical use, and besides comparative studies, a huge amount of real life data showed their substantial equivalence in inducing multiple follicular growth, oocyte yield, and live birth rate after IVF [39,40].

Follitropin *δ* was introduced on the market much more recently; its efficacy was compared to follitropin *α* in the phase III study ESTHER-1, including 1326 women submitted to COS using either a starting dose of follitropin *δ* based on body weight and anti-Müllerian hormone (AMH) or a daily dose of 150–450 IU of follitropin *α* [41,42]. The non-inferiority of follitropin *δ* to follitropin *α* for the primary endpoints (ongoing pregnancy rate: 30.7% and 31.6%, respectively, and ongoing implantation rate: 35.2% and 35.8%, respectively) was demonstrated. Also the live birth rate was similar between follitropin *α* and follitropin *δ* (29.8% and 30.7%, respectively), and fewer women in the follitropin *δ* study arm required preventive measures against ovarian hyperstimulation syndrome (OHSS) [41]. The European Medicines Agency (EMA) assessment report stated that in the ESTHER-1 trial, the non-inferiority of follitropin *δ* vs. follitropin *α* was affected by the heterogeneity of response in different age groups. Indeed, Lunenfeld et al. reported that the non-inferiority was applied to the 15% of the study population aged ≥38 years, whereas it was not demonstrated for women aged ≤37 [34]. Finally, it was also noted that there was a higher number of cancelled cycles due to poor response in the follitropin *δ* arm [41]. The risk/benefit balance of follitropin *δ* was considered positive, as the most frequent adverse reactions reported were headache, pelvic discomfort, nausea, and fatigue [23]. Follitropin *δ* demonstrated higher bioavailable dose and lower serum clearance compared with follitropin alfa [25]. Based on these differences, it was concluded that follitropin *δ* and *α* are not easily interchangeable in clinical practice.

After a secondary analysis of ESTHER-1, Fernández-Sánchez et al. confirmed that individualized dosing with follitropin *δ* significantly reduced moderate/severe OHSS and/or the need of OHSS preventive interventions in patients undergoing up to three COS cycles, and the greatest benefit was observed in patients in the highest AMH quartile, those with the maximal risk of severe OHSS [43].

Another study, the controlled, assessor-blind ESTHER-2 showed that in women undergoing repeated COS cycles (cycles 2 and 3) following initial stimulation with follitropin *δ* or *α* (cycle 1), the incidence of treatment-induced anti-hFSH antibodies with follitropin *δ* was 0.8% and 1.1% in cycles 2 and 3, respectively, which was similar to the incidence in cycle 1 (1.1%) [44]. Treatment with either follitropin *δ* or *α* obtained similar mean number of retrieved oocytes (9.2 versus 8.6 (cycle 2); 8.3 versus 8.9 (cycle 3)), ongoing pregnancy rate (27.8% versus 25.7%; 27.4% versus 28.0%) and live birth rate (27.4% versus 25.3%; 26.3% versus 26.9%). Notably, women with pre-existing anti-hFSH antibodies were safely treated with follitropin *δ* without boosting an immune response or affecting the ovarian response, suggesting a lack of immunogenicity of follitropin *δ* in patients undergoing repeated ovarian stimulation cycles, similarly to follitropin *α* and follitropin *β* that had not shown any anti-hFSH antibody production [45]. 

In comparative clinical trials, CF*α* was shown to be an effective alternative to daily injections of r-hFSH in COS [46]. Comparable results in terms of ongoing pregnancy rate, miscarriage rate, and live birth rate were reported with the use of CF*α* versus daily r-hFSH [47]. However, an increased risk of OHSS in patients defined as high responders (e.g., young women with polycystic ovary) was also reported [47], limiting the use of CF*α* to specific cohorts of IVF patients, those with an expected normal or poor ovarian responsiveness to r-hFSH. 

To date, no phase III studies have been registered in available clinical trial repositories for follitropin ε.

## 5. Biosimilar r-hFSH (Follitropin *α*)

The term “biosimilar” describes an off-patent copy of a therapeutic substance [48]. The US Food and Drug Administration (FDA) characterizes biosimilars as biologic products that are highly similar to the reference product (originator), notwithstanding minor differences in clinically inactive components, and that have no clinically relevant differences in comparison with the reference product in terms of safety and efficacy [49]. This definition describes a product that is similar, but not identical to the originator. Indeed biosimilars may still differ from the originator in biological potency, purity, composition of isoforms and/or various glycosylation profiles [50], with consequent differences in the clinical efficacy and/or safety [51,52]. This “non-identity” represents the main difference from generic drugs, which are small synthetic molecules chemically identical and fully bioequivalent to the brand listed medication, identical both in pharmacokinetic and pharmacodynamic characteristics [53]. 

As biosimilarity is a concept alluding to the evidence-based high-standard comparability, studies are needed to demonstrate the equivalence of a biosimilar to the originator. Differently from chemically-synthesized medications, biotechnology-derived products are subject to an inherent molecular variability, even if minor batch-to-batch physicochemical variations may be therapeutically acceptable [48].

The patent of follitropin *α* expired in 2012 in many European countries [54], and it became possible to produce a biosimilar follitropin *α*. To date, two follitropin *α* biosimilars, Ovaleap^®^ (Theramex Ireland Limited, Dublin, Irland) and Bemfola^®^ (Afolia^®^, Finox Biotec AG, Balzers, Liechtenstein) have been authorized by the EMA. The first is produced by a CHO-derived cell line after adaptation to serum free conditions [55], whereas the second is produced by a pre-adapted dihydrofolate reductase deficient CHO host cell line [56]. Both follitropin *α* biosimilars are administered via the same route, at the same dose, and for the same indications [53]. Their production is strictly regulated by EMA guidelines, and their use has been approved in the USA since 2016 [57]. 

Moreover, some other r-hFSH biosimilars have been marketed only in their countries of origin, such as Primapur^®^ (IVFarma LLC, Moscow, Russia), DA-3801^®^ (Dong-A ST/Genexine, Seoul, South Korea), Folitime^®^ (also named GEMA, Amega Biotech S.A., Buenos Aires, Argentina), LM-001^®^ (also named Alphamab, Jiangsu Alphamab Biopharmaceuticals Co. Ltd, Andhra Pradesh, India), Gonapure^®^ (Minapharm Pharmaceuticals, Cairo, Egypt) and Cinnal-F^®^ (CinnaGen, Tehran, Iran and Singapore Biotech, Singapore).

Winstel et al. compared the originator Gonal-F^®^ to the biosimilar Ovaleap^®^ for the molecular mass, primary and secondary structure, in vitro biological activity, long-term stability at room temperature and product-related impurities, demostrating that biosimilar and originator have similar characteristics derived from the manufacturing process [55]. Moreover, increasing concentrations of the originator or of its biosimilars were used for inducing primary granulosa luteinization and hFSH receptor-transfected human embryonic kidney (HEK293) cellular responses [13]. Ricetti et al., comparing the originator to the biosimilars, demonstrated that they induced similar intracellular responses and steroidogenesis in HEK293 cells, reflecting similar bioactivity, and overall structural homogeneity. Slight differences in glycosylation profiles characteristic of the follitropin *α* were detected and are likely due to the specific enzymatic equipment of the source cell lines [13]. 

Regarding the molecular mechanisms underlying hFSH activity on steroidogenesis, proliferation and survival/apoptosis, several studies focused on the Gs/cAMP/PKA pathway activated by hFSH after binding to its receptor [58]. Follitropin *α* originator and biosimilars revealed comparable in vitro hormone-induced intracellular signalling and effect on steroidogenesis, resulting in similar dose-response curves for both the 3′,5′-cyclic adenosine monophosphate (cAMP) synthesis, enhanced through the response element binding protein (CREB) and extracellular-regulated kinase 1/2 (ERK1/2) phosphorylation [59], and the intracellular Ca^2+^ increase [60]. 

## 6. Clinical Trials Comparing Follitropin *α* Biosimilars vs. Originator 

After manufacturing of the biosimilar product a phase III Randomised Clinical Trial (RCT) is required to demonstrate that structural changes do not adversely affect the identity, purity, or potency of the potentially approved biologic product [61].

The first follitropin alpha biosimilar approved for clinical use in Europe was Ovaleap^®^, that was approved by EMA in 2013 [62] basing on the clinical trial NCT02809989 (Table 1). To establish the efficacy of Ovaleap^®^ compared to the follitropin *α* originator Gonal-F^®^, EMA requested a single prospective randomized trial in which the primary endpoint was the number of retrieved oocytes; as for the safety, the incidence of OHSS was the main considered indicator [61]. Equivalence was considered as demonstrated if the two-sided 0.95 confidence interval for the difference in the number of retrieved oocytes would have fallen within the range of ± 3 oocytes. The phase III study was performed by Strowitzki et al., who studied a selected population of women undergoing COS for IVF and found that Ovaleap^®^ was equivalent to Gonal-F^®^ in terms of retrieved oocytes [63]. This multinational, multicenter, randomized (1:1), active-controlled, assessor-blind, comparative study included infertile women aged 18-37, with a body mass index between 18 and 29 kg/m^2^ and regular menstrual cycles of 21 to 35 days. During the initial 5-day fixed-dose phase, 153 women received 150 IU/day of Ovaleap^®^, and 146 received the same dose of Gonal-F^®^; a 10–15-days dose-adaptation phase followed, during which the administered dose could be adjusted every 3–5 days, up to a maximum of 450 IU/day. Using an imputation value of zero for patients without oocyte retrieval, Ovaleap^®^-treated patients obtained 12.2 ± 6.8 retrieved oocytes vs. 11.9 ± 6.9 for Gonal-F^®^–treated patients. Without applying any imputation, oocyte retrieval was nearly identical in either arms (12.2 ± 6.7 vs. 12.1 ± 6.7 with Ovaleap^®^ and Gonal-F^®^, respectively). In the same study, several secondary endpoints were considered: the follicle number, size, and the endometrial thickness resulted to be comparable between groups; approximately 90% of the started clinical pregnancies ended with a live birth, 89.1% (41/46) in the Ovaleap^®^ group and 88.7% (47/53) in the Gonal-F^®^ group. The take-home baby rates, defined as the percentage of randomized patients whose treatment cycle ended with a live birth, were comparable: 26.8% (41/153) with Ovaleap^®^ and 32.2% (47/146) with Gonal-F^®^, respectively. Among undesired effects, the OHSS rate was 4.6% (7/153) in the Ovaleap^®^ group and 2.7% (4/146) in the Gonal-F^®^ group, abdominal pain was observed in 3.3% of patients (5/153) treated with Ovaleap^®^ and 0.7% (1/146) of those treated with Gonal-F^®^ [63]. Finally, Strowitzki et al. concluded that Ovaleap^®^ had shown the same efficacy and safety as Gonal-F^®^ for ovarian stimulation of infertile women below 37 years undergoing IVF. 

The second European biosimilar follitropin *α*, Bemfola^®^ (Afolia^®^), was approved by EMA in 2014 [56]. Preliminarly, in a phase I, randomized, open-label, crossover trial (NCT02459418), Wolzt et al. showed in 32 healthy young women that Bemfola^®^ exhibited clinical pharmacokinetic and safety profiles comparable to Gonal-F^®^ [64]. The clinical trial for its approval on market was a randomized, multicentre, phase III study (NCT01121666) (Table 1) including 372 women undergoing IVF, in which the efficacy and safety of Bemfola^®^ was compared to that of the originator Gonal-F^®^ [65]. A selected population of women aged 20–38 were randomized 2:1 to receive a single, daily, subcutaneous 150 IU dose of either Bemfola^®^ or Gonal-F^®^. The study primary endpoint was the number of retrieved oocytes; equivalence was considered as demonstrated if a difference lower than ± 2.9 oocytes would have been observed. Bemfola^®^ and Gonal-F^®^ treatments resulted in a comparable number of retrieved oocytes (10.8 ± 5.11 vs. 10.6 ± 6.06, respectively); among the secondary endpoints, a similar clinical pregnancy rate per embryo transfer in the first and second treatment cycles was observed (Bemfola^®^: 40.2% and 38.5%, respectively; Gonal-F^®^: 48.2% and 27.8%, respectively). The incidence of OHSS was 5.6% in the Bemfola^®^ group and 3.3% in the Gonal-F^®^ group. Thus, Rettenbacher et al. [65] concluded that Bemfola^®^ and Gonal-F^®^ had similar clinical efficacy and safety profiles. 

Unfortunately, these two clinical trials appear to have some major weak points: (1) they were both performed on a selected population of young women undergoing IVF, with an expected optimal responsiveness to r-hFSH, and (2) their power calculation was performed choosing the number of retrieved oocytes as the primary endpoint, and leaving other clinically pivotal variables (e.g., ongoing pregnancy rate, live birth rate) as secondary endpoints [61]. Indeed, the average population of women undergoing IVF in the daily routine clinical practice includes a relevant proportion of subjects older than 37, with an expected reduced responsiveness to COS and needing a starting dose much higher than 150 IU/day, which was the dose used in registration trials. Moreover, in the real-life clinical practice several women undergoing IVF have PCOS, with irregular, anovulatory cycles, hyperandrogenism, and a clear tendency at overresponding to FSH, developing a huge number of middle-size growing follicles and having a risk of OHSS much higher than average. A clinical study aimed at assessing whether a medication is suitable for COS should include a wide population mirroring the one that is usually encountered in the daily work, and not a selected, ideal population of subjects with better-than-average prognosis and lower-than-average risk. 

It is quite common that studies dealing with medications used in COS adopt the number of retrieved oocytes as the primary endpoint representing a sensitive endpoint for an accurate comparison between such medications [53], as it avoids differences that may not be attributable to the product. Indeed ongoing pregnancy rate or live birth rate could be linked to confounding factors unrelated to medications, and should be rather used as a secondary efficacy measure [53]. However, this view is questionable for at least two reasons. 

First, there is no doubt that the effectiveness of IVF is witnessed by live births, and that live birth rate is the only endpoint fully expressing the efficacy of any infertility treatment. When two procedures (e.g., two different incubators for human embryos) are compared in an RCT, the only acceptable primary endpoint is live birth rate, and no surrogate endpoints (e.g., the proportion of embryos surviving the culture) are accepted to establish equivalence. The reason why in some clinical RCTs dealing with medications the primary endpoint is the number of retrieved oocytes is only economical: this choice allows to include in the study a rather low number of patients (as all women produce several oocytes), whereas choosing the live birth as primary endpoint would compel to include many more observations (only about 30% of women will have a live birth), increasing relevantly the cost of the trial.

Second, when a study is powered according to a given primary endpoint, comparing the secondary endpoints must be considered just an occasional observation; drawing conclusions after comparing the secondary endpoints is meaningless and incorrect, as the number of observations is insufficient to get a meaningful comparison. In other words, if a study comparing two drugs is powered to detect a difference in the number of retrieved oocytes, no meaningful comparison can be made for the number of pregnancies, live births, or for the incidence of OHSS: these are just occasional observations that should be verified in further, adequately powered studies.

Analyzing the two registration trials, other points were also noticed. The increased rate of OHSS observed with Bemfola [66] could be due to a more variable and abrupt oestradiol rise, in turn linked to a higher batch-to-batch variability and to differences in glycosylation profiles [66]. Indeed, even if Bemfola^®^ and Ovaleap^®^ are similar to the reference product Gonal-F^®^, their structures are analytically not identical due to post-translational modifications that result from differences in the production and purification systems [34]. Specifically, differences in glycosylation were observed between the biosimilars and Gonal-F^®^, with Bemfola^®^ showing higher antennarity, higher sialylation and higher batch-to-batch variability in activity compared to Gonal-F^®^ [66], whereas Ovaleap was shown to have a higher amount of the sialic acid N-glycolyl neuraminic acid compared with Gonal-F^®^ [53]. These differences were considered by EMA as minor and acceptable, even if they could result in more pronounced variability in hFSH receptor activation [66]. These findings could be of clinical importance especially in “non-ideal” patients, like older women poorly responding to r-hFSH, or, on the other side, young women with PCOS and subsequent “constitutional” hyper-responsiveness to COS [61].

In another clinical trial, the efficacy of Bemfola^®^ was compared with that of a HP-uhFSH (Fostipur^®^, IBSA, Farmaceutici Italia Srl, Lugano, Switzerland). Requena et al. performed a phase IV randomized, parallel-group, trial (NCT02503605) including 130 oocyte donors aged 18–35 years, with BMI 18–30 kg/m^2^ and antral follicle count (AFC) >20, suggesting an optimal ovarian responsiveness to COS. The number of retrieved, mature oocytes was considered the primary outcome whereas days of stimulation, total FSH dose, estradiol and progesterone concentration at ovulation trigger, fertilization rate, number of cryopreserved embryos, implantation rate, cancellation rate and patient compliance were all considered as secondary outcomes. The study was started in 2016, but no preliminary data are available yet [67]; of note, this study has the same conceptual limitations of the previous ones, which are the choice of a selected, optimal population, and a power calculation based on a primary endpoint that does not fully express IVF efficacy.

Other observational, phase III and IV studies on Bemfola^®^ that are still ongoing or have been completed but have not yet published the results, are shown in Table 1.

Recently, after the NCT03857230 (Table 1) phase I interventional trial demonstrating the safety of the follitropin *α* biosimilar Primapur^®^, a multicenter, randomized (1:1), embryologist-blinded, parallel-group, comparative phase III study (NCT03088137) was performed (Table 1) [68]. It enrolled good prognosis women aged 20–35 years with tubal and/or male infertility, who underwent COS using a gonadotropin-releasing hormone antagonist (GnRH-ant) protocol. Over the initial 5-day fixed-dose regimen, patients received 150 IU/day of Primapur^®^ (*n* = 55) or Gonal-F^®^ (*n* = 55), followed by dose adaptation. Again, the primary endpoint for assessing the therapeutic equivalence was the number of retrieved oocytes, using a pre-determined clinical equivalence margin of ±3.4 oocytes. A similar number of oocytes was retrieved in both groups (12.16 ± 7.28 in the Primapur^®^ group and 11.62 ± 6.29 in the Gonal-F^®^ group). Additionally, no statistically significant differences were found for secondary endpoints: clinical pregnancy rate (26.5% vs. 32.7%, respectively), and take-home baby rate (28.6% and 26.5%, respectively). OHSS was observed in 7.27% and 3.64% of patients in the biosimilar and originator groups, respectively.

The safety and efficacy of the follitropin alpha biosimilar DA-3801 was investigated by Moon et al. [69] in a phase III, multicenter, randomized, non-inferiority trial (NCT01820728) comprising 97 women randomized to receive COS using DA-3801 (*n* = 49) or Gonal-F^®^ (*n* = 48) (Table 1). The number of retrieved oocytes was set as primary endpoint, whereas the total FSH dose, the length of stimulation, the serum estradiol levels on the day of oocyte maturation trigger, and the fertilization, implantation and pregnancy rates were considered as secondary endpoints. The number of retrieved oocytes was 13.0 ± 6.2 versus 10.6 ± 6.7 for DA-3801 and Gonal-F^®^, respectively, in the intention-to-treat (ITT) population, and 12.7 ± 6.4 versus 11.0 ± 7.1 for DA-3801 and Gonal-F^®^, respectively, in the per-protocol (PP) population. The non-inferiority of DA-3801 was assessed with differences of 2.3 ± 6.5 and 1.7 ± 6.7, in the ITT and PP populations, respectively. The total dose of FSH (1789.8 ± 465.5 versus 2055.6 ± 646.7 pg/mL) and the length of stimulation (8.3 ± 1.4 versus 9.1 ± 1.9 days) in the ITT population were significantly lower in the DA-3801 group. Pregnancy and implantation rates, as well as the incidence of OHSS, were comparable in the two groups. The previously reported criticism on the study methodology applies also in these two studies. Other clinical trials for other biosimilars of follitropin *α* (Folitime^®^, LM-001^®^ and GONAPUR^®^) have recently been concluded, but the results are not yet available (Table 1).

## 7. Cost-Effectiveness Evaluation of Follitropin *α* Biosimilars vs. Originator in the European Context

A cost-effectiveness evaluation comparing the originator follitropin *α* Gonal-F^®^ to the biosimilar Bemfola^®^, based on data derived from the registration study by Rettenbacher [65], established that the originator r-hFSH is more cost-efficient than the biosimilar relative to the conditions of medical reimbursement existing in Italian and Spanish health systems. All clinical data and the costs of the procedure, including the overall r-hFSH dose and the costs related to drugs, hospitalizations and examinations were taken into account and related to the number of live births; compared to the originator, the biosimilar generated an higher cost of € 3600 for Italy and € 900 for Spain [70].

In the French context with National Health Service perspective, the average increased cost per live birth was € 259.56 and € 278.39, respectively, for Ovaleap^®^ and Bemfola^®^ vs. Gonal-F^®^ that resulted to be cost-effective compared to its biosimilars [71].

Moreover, the cost-effectiveness of Gonal-F^®^ in comparison to Bemfola^®^ and Ovaleap^®^ from a German payer perspective was reported. Results indicated that the average cost per live birth for women treated with the originator was lower: Bemfola^®^ and Ovaleap^®^ were associated with a higher cost of € 4168 and € 7540, respectively, per additional live birth [72].

Concerning the cost/effectiveness of biosimilar FSH preparations, further studies are needed, extending the investigation also to other countries with different systems of public health economy where biosimilars are on the market.

## 8. Conclusions

Interestingly enough, the worldwide web-based survey [73], showed that most IVF Centers (67.3%) are aware of the availability of FSH biosimilars on the market, but 90% of them require more complete information on these products. This limits the clinical use of biosimilars, as a relatively low number of centres (25.6%) reported to have clinical experience with these new products.

As a matter of fact, the two follitropin *α* biosimilars already authorized and marketed as Ovaleap^®^ and Bemfola^®^ were compared to the reference product Gonal-F^®^ in a few studies performed on selected, good prognosis patients, and powered to detect a difference in the number of retrieved oocytes. The most important endpoints of IVF, the live birth rate and the incidence of OHSS, were considered just secondary endpoints and the inadequate number of observations did not allow reaching any meaningful conclusion about the equivalence of the biosimilars to the originator with their respect.

Moreover, the canonical G_s_/cAMP/protein kinase A pathway, considered for a long time as the sole effector of the hFSH receptor-mediated signaling, is now viewed just as one of several mechanisms employed by hFSH receptor to transduce intracellular signals in response to the stimulus [74]. The complexity of the hFSH receptor-mediated intracellular signals activated in response to ligand binding allows for a fine-tuning regulation of the gonadotropic stimulus, where activation/inhibition of its multiple components vary depending on the cell context, cell developmental stage, concentration of associated receptors and, above all, corresponding ligands. For this reason, and in light of the different glycosylation profiles of biosimilars, linked to source and/or purification process [13,66], other signaling pathways may be involved in determining r-hFSH action and potentially may cause subtle differences in the final outcome after clinical use of r-hFSH medications.

Post marketing, real-world data studies and pharmacovigilance data are definitely needed to assess whether follitropin *α* biosimilars really have comparable clinical efficacy to the originator, expanding observation also to “non-ideal” patients and reaching a much higher number of observations. To date, despite promising data, there is no proof yet of the equivalence of r-hFSH biosimilars with their originator in the real-life patient population undergoing IVF and with respect to the only endpoint that really matters, which is live birth rate and OHSS incidence. Definitely, further well designed and properly powered trials are needed in this area.

## Figures and Tables

**Table 1:** Clinical trials available in the literature and in the U.S. National Library of Medicine|U.S. National Institutes of Health|U.S. Department of Health & Human Services (available online at: https://clinicaltrials.gov/ct2/home) considering follicle-stimulating hormone (FSH) biosimilar administration to women for infertility treatment.

Drug Name	NCT Number	Title	Status/Country	Study Results	Study Design	Outcome Measures
**OVALEAP^®^**	NCT02809989	A study to evaluate the effect of Ovaleap^®^ on the pregnancy rate and clinical effects as well as the user-friendliness of the Ovaleap^®^-Pen	Completed in 2018 Germany	Results available [63]	**Study Type**: Observational **Observational model**: Single group prospective treatment cohort **Enrolment**: 507 participants (18–40 years) **Drug**: Ovaleap^®^	Number of retrieved oocytes (primary) Clinical pregnancy rate (primary) Days of stimulation Total Ovaleap^®^ dose administered Estradiol at the ovulation trigger Endometrial thickness at the ovulation trigger Type of ovulation trigger (*β*-hCG, GnRH agonist) Number of metaphase II (MII) oocytes Number of fertilised oocytes Day of embryo transfer and number of transferred embryos and 5 more
**AFOLIA^®^**	NCT02459418	Comparative pharmacokinetics of AFOLIA and US Gonal-F^®^ RFF redi-ject after single subcutaneous application	Completed in 2016 United Kingdom	Results available [64]	**Study Type**: Interventional **Phase**: Phase I **Allocation**: Randomized **Intervention model**: Crossover assignment **Masking**: None (Open Label) **Enrolment**: 42 participants (18-42 years) **Drugs**: Afolia, Gonal-F^®^	Baseline corrected FSH area under the serum concentration-time curve from zero to the last quantifiable measurement (AUC (0-last)) (primary) Baseline corrected FSH maximum serum concentration (Cmax) (primary) Baseline corrected FSH area under the serum concentration-time curve extrapolated to infinity (AUC (0–∞)) Baseline corrected time to reach maximum FSH serum concentration (Tmax) Baseline corrected FSH apparent terminal half-life Baseline corrected 17ß-estrodiol (E2) serum exposure AUC (0-last) Baseline corrected E2 Cmax Baseline corrected E2 Tmax
**FOLIA^®^**	NCT01687712	Phase III study comparing efficacy and safety of AFOLIA vs. Gonal-F^®^ RFF in women (35 to 42) undergoing IVF	Completed in 2016 United States	Results available, not published yet	**Study Type**: Interventional **Phase**: Phase III **Allocation**: Randomized **Intervention model**: Parallel Assignment **Masking**: Double (Investigator, Outcomes Assessor) **Enrolment**: 1100 participants (35–42 years) **Drugs**: Afolia, Gonal- F^®^	Clinical pregnancy rate after one cycle of treatment - ITT population (primary) Clinical pregnancy rate after one cycle of treatment - PP Population (primary) Days of stimulation-cycle 1 Total r-hFSH dose administered - cycle 1 Daily r- hFSH dose-cycle 1 Number of retrieved oocytes-cycle 1 Local and systemic adverse events: dermal response to injection-cycle 1 Local and systemic adverse events: dermal response to injection by severity-cycle 1 Overall summary of adverse events (AEs)-cycle 1 Adverse events of special interest: and 5 more
**AFOLIA^®^**	NCT01141270	Comparative pharmacokinetics of AFOLIA and Gonal-F^®^	Completed in 2010 Austria	No results available	**Study Type**: Interventional **Phase**: Phase I **Allocation**: Randomized **Intervention model**: Crossover assignment **Masking**: None (Open Label) **Enrolment**: 32 participants (18–38 years) **Drugs**: Afolia, Gonal-F^®^	Area under the serum concentration curve (AUC) of FSH (primary)
**AFOLIA^®^**	NCT01121666	Multi-centre study to compare efficacy and safety of AFOLIA and Gonal-F^®^ in women	Completed in 2013 Austria, Germany	Results available [65]	**Study Type**: Interventional **Phase**: Phase III **Allocation**: Randomized **Intervention model**: Parallel Assignment **Masking**: Single (Outcomes Assessor) **Enrolment**: 460 participants (20–38 years) **Drugs**: Afolia, Gonal- F^®^	Number of retrieved oocytes (per protocol population) (primary) Number of retrieved oocytes (intention-to-treat population) (primary) Number and size of follicles ≥12 mm on day 8 of stimulation E2 concentration on day 8 and the day of ovulation trigger Total r-hFSH dose administered Oocyte quality Fertilisation rate Embryo quality: mean number of blastomeres Number of participants with cryopreserved 2PNs, embryos/blastocysts Days of stimulation and 9 more
**BEMFOLA^®^**	NCT02942849	Post-authorisation study on the use of Bemfola^®^ in human assisted reproductive technology	Completed in 2018 Germany	No results available	**Study Type**: Observational **Observational model**: cohort, prospective **Enrolment**: 1195 participants (≥18 years) **Drug**: Bemfola^®^	Number of retrieved oocytes (primary) Antral follicle count (AFC) Basal FSH level r-hFSH dose on first and last day of stimulation Days of stimulation Total r-hFSH dose administered Type of ovulation trigger (ß-hCG, GnRH agonist Number of fertilised oocytes Number of cryopreserved 2PN embryos Number of transferred embryos and 5 more
**BEMFOLA^®^**	NCT03767218	Ignition of ovarian stimulation with recombinant human FSH (Bemfola) in the late follicular phase	Recruitment completed in 2020	No results available	**Study Type**: Interventional **Phase**: Phase III **Allocation**: Randomized **Intervention model**: Two-arm design with 1:1 **Masking**: None (Open Label) **Enrolment**: 40 participants (18–36 years) **Drug**: Bemfola^®^	Number of COC (cumulus-oocyte-complex) (primary) Endocrine profile Total r-hFSH dose administered Days of stimulation Days of GnRH antagonist Number of cryopreserved oocytes
**BEMFOLA^®^**	NCT02941341	Observational post- authorisation study on the use of Bemfola^®^ in human assisted reproductive techniques in Spain	Completed in 2020 Germany	No results available	**Study Type**: Observational **Observational model**: cohort, prospective **Enrolment**: 1222participants (≥18 years) **Drug**: Bemfola^®^	Number of retrieved oocytes (primary) Number of fertilised oocytes Embryo quality Number and quality of transferred embryos Fertilisation and implantation rate Incidence of serious adverse events, including moderate-to-severe OHSS
**BEMFOLA^®^**	NCT02503605	Biosimilar versus urinary gonadotropins study documents	Unknown Spain	No results available	**Study Type**: Interventional **Phase**: Phase IV **Allocation**: Randomized **Intervention model**: Parallel Assignment **Masking**: None (Open Label) **Enrolment**: 130 participants (18–35 years) **Drugs**: Bemfola^®^, urinary FSH	Number of metaphase II (MII) oocytes (primary) Days of stimulation Total FSH dose administered Estradiol at the ovulation trigger Progesterone at the ovulation trigger Fertilisation rate Percentage of cryopreserved embryos Implantation rate Cancellation rate Degree of satisfaction (numbers 0-10) Apoptosis rate in granulosa cells
**BEMFOLA^®^**	NCT02625519	Efficacy of urinary vs. recombinant FSH in oocyte donors based on receptor N680S FSH gene polymorphism (genodon trial)	Completed in 2019 Spain	No results available	**Study Type**: Interventional **Phase**: Phase IV **Allocation**: Randomized **Intervention model**: Parallel Assignment **Masking**: None (Open Label) **Enrolment**: 180 participants (18-30 years) **Drugs**: Bemfola^®^, urinary hFSH,	Number of COC (cumulus-oocyte-complex) obtained (primary) Number of COC (cumulus-oocyte-complex) obtained/puncture (primary) Number of metaphase II (MII) oocytes Number of inseminated/microinjected oocytes Days of stimulation FSH treatment units obtained by oocyte FSH treatment cost per oocyte obtained Fertilisation rate Occurrence of side effects
**FOSTIPUR^®^**	NCT02785822	Study to Compare hFSH- HP (Fostipur) and hMG HP (Meriofert) in patients with polycystic ovary under a IVF/CSI cycle	Completed in 2018 Spain	No results available	**Study Type**: Interventional **Phase**: Phase IV **Allocation**: Randomized **Intervention model**: Parallel assignment **Masking**: Single (Investigator) **Enrolment**: 19 participants (18–38 years) **Drugs**: Fostipur^®^, hMG-HP	Number of metaphase II (MII) oocytes with respect to the total oocytes (primary)
**PRIMAPUR^®^**	NCT03857230	The safety and pharmacokinetics of Primapur^®^ and Gonal-F^®^	Completed in 2019 Russia	Results available [68]	**Study Type**: Interventional **Phase**: Phase I **Allocation**: Randomized **Intervention model**: Crossover Assignment **Masking**: None (Open Label) **Enrolment**: 28 participants (18–40 years) **Drugs**: Primapur^®^, Gonal- F^®^	Area under the serum concentration of FSH - Time Curve (AUC (0-192)) (primary) Maximum serum concentration of FSH (Cmax) (primary) Time to reach a maximum FSH serum concentration (Tmax) FSH apparent terminal half-life (T1/2)
**PRIMAPUR^®^**	NCT03088137	Study to compare efficacy and safety of Primapur^®^ and Gonal-F^®^ in women for assisted reproductive treatment	Completed in 2018 Russia	Results available [68]	**Study Type**: Interventional **Phase**: Phase III **Allocation**: Randomized **Intervention model**: Parallel Assignment **Masking**: Single (Outcomes Assessor) **Enrolment**: 118 participants (20–35 years) **Drugs**: Primapur^®^, Gonal- F^®^	Number of oocytes (Intention-to-Treat, ITT) (primary) Number of follicles with size ≥16 mm Number of metaphase II (MII) oocytes Number of fertilised oocytes Percentage of patients with Embryo Transfer Total FSH dose administered Days of stimulation Number of patients with FSH dose correction Number of patients with cycle cancellation Number of no-responders Pregnancy rate Clinical pregnancy rate
**DA-3801^®^**	NCT01820728	A Phase III clinical study to compare the efficac and safety of DA-3801 and that of Gonal-F^®^	Completed in 2012 Korea	Results available [69]	**Study Type**: Interventional **Phase**: Phase III **Allocation**: Randomized **Intervention model**: Parallel Assignment **Masking**: None (Open Label) **Enrollment**: 93 participants (20–38 years) **Drugs**: DA-3801, Gonal-F^®^	Ovulation rate after 3 cycles (primary) Total FSH dose administered Days of stimulation Threshold dose, IU Number of follicles
**FOLITIME^®^**	NCT02454556	A randomized, multicentre, open label, evaluator blinded study to evaluate safety and efficacy of Folitime^®^ of Gemabiotech S.A. versus Gonal-F^®^ of Merck Serono, in patients with infertility undergoing ART	Completed in 2016 Argentina	No results available	**Study Type**: Interventional **Phase**: Phase III **Allocation**: Randomized **Intervention model**: Parallel Assignment **Masking**: Single (Outcomes Assessor) **Enrollment**: 106 participants (18–37 years) **Drugs**: FOLITIME^®^, Gonal-F^®^	Number of retrieved (primary)
**LM-001^®^**	NCT03535103	Study on the safety and pharmacokinetics of LM001 and Gonal-F^®^ in healthy women	Unknown Argentina	No results available	**Study Type**: Interventional **Phase**: Phase I **Allocation**: Randomized **Intervention model**: Crossover Assignment **Masking**: None (Open Label) **Enrollment**: 32 participants (18–40 years) **Drugs**: LM001, Gonal-F^®^	Maximum observed serum concentration (Cmax) of LM001 & Gonal-F^®^ (primary) Adjusted geometric means of area under the serum concentration-time curve from time zero to the time of last quantifiable concentration (AUC(0-T)) for LM001 & Gonal-F^®^ (primary) Time of Maxmum observed serum concentration (Tmax) of LM001& Gonal-F^®^
**GONAPUR^®^**	NCT03057574	Gonapure^®^ in multifollicular stimulation in Egyptian women undergoing IVF/ICSI	Unknown Egypt	No results available	**Study Type**: Interventional **Phase**: Phase 4 **Allocation**: N/A Intervention model: Single Group Assignment **Masking**: None (Open Label) **Enrollment**: 200 participants (18–38 years) **Drug**: Gonapure^®^	Number of retrieved oocytes (primary) Number of metaphase II (MII) oocytes (primary) Oocyte quality Eventual AE/SAEs related to the administration of Gonapure^®^ Total and mean Gonapure ^®^ dose administered Number of follicles ≥18 mm at the ovulation trigger Multiple pregnancy rate OHSS rate
